# Effects of glycopyrrolate and atropine for oral secretions and perioperative hemodynamics in children undergoing tonsillectomy and adenoidectomy: a prospective, single-center, randomized, double-blind, controlled trial

**DOI:** 10.3389/fphar.2024.1344786

**Published:** 2024-05-09

**Authors:** Yi-Bin Tao, Zi-Li Tang, Zhong-Lan Lin, Wei-Ping Lei, Xin-Lei Lu, Jian-Liang Sun

**Affiliations:** ^1^ Department of Anesthesiology, The Fourth Clinical School of Medicine, Zhejiang Chinese Medical University, Hangzhou, China; ^2^ Department of Anesthesiology, Affiliated Hangzhou First People’s Hospital, Westlake University School of Medicine, Hangzhou, China

**Keywords:** glycopyrrolate, tonsillectomy, adenoidectomy, pediatrics, oral secretions, general anesthesia, hemodynamics

## Abstract

**Introduction:**

Glycopyrrolate is commonly researched as a preoperative medication or in conjunction with cholinesterase inhibitors to counteract the lingering muscarinic effects of non-depolarizing muscarinic agents. However, studies have yielded inconsistent results regarding the superiority of glycopyrrolate over other anti-cholinergic drugs, such as atropine, particularly its effect on heart rate, blood pressure (BP), and glandular secretions. This study aimed to evaluate the differences in perioperative oral secretions, hemodynamics, and recovery quality with glycopyrrolate versus those with atropine before anesthesia induction in children undergoing tonsillectomy and adenoidectomy.

**Methods:**

In this prospective, single-center, randomized, double-blind, controlled trial, a total of 103 children were randomly assigned to group A (n = 51, glycopyrrolate 0.005 mg/kg) or B (n = 52, atropine 0.01 mg/kg). The follow-up anesthetic induction and maintenance protocols were the same in both groups. Vital signs, duration of surgery, extubation time, degree of wetness around the vocal cords during tracheal intubation, weight of oral secretions, and perioperative complications were recorded.

**Results:**

No significant differences were observed in the degree of wetness around the vocal cords during tracheal intubation, as well as in the weight of oral secretions, duration of surgery, or extubation time, between the two groups. The intraoperative and postoperative heart rates were lower in group A than in group B (110.18 ± 10.58 vs. 114.94 ± 11.14, *p* = 0.028; 96.96 ± 10.81 vs. 103.38 ± 10.09, *p* = 0.002). The differences observed in the intraoperative and preoperative heart rates were lower in group A than in group B (23.84 ± 9.62 vs. 29.65 ± 8.75, *p* = 0.002). The differences observed in the postoperative and preoperative heart rates were lower in group A than in group B (10.63 ± 9.97 vs. 18.09 ± 9.39, *p* = 0.000).

**Conclusion:**

Glycopyrrolate showed a smoother change in heart rate than atropine during and after tonsillectomy and adenoidectomy, with no effect on BP or recovery quality, and did not increase oral secretions. The findings indicate that glycopyrrolate can serve as an alternative to atropine to prevent secretions in anesthesia induction for tonsillectomy and adenoidectomy in children.

**Trial registration:** This study was registered with the Chinese Clinical Trial Registry (Registration Number: ChiCTR2200063578; Date of Registration: 12/09/2022).

## Introduction

Chronic tonsillitis and adenoid hypertrophy are common conditions in pediatric otolaryngology and typically manifest with symptoms such as breathlessness, sleep snoring, swallowing difficulties, and speech resonance disorders (Sun and Zhang, 2018). General anesthesia with tracheal intubation is a widely used and effective procedure in tonsillectomy and adenoidectomy. In pediatric patients, glandular secretions pose a significant concern and could cause complications such as hypoxemia, airway obstruction, laryngospasm, and other incidents. Therefore, managing perioperative airway secretions is a critical consideration when performing general anesthesia in children. The administration of intravenous anti-cholinergic drugs preoperatively can effectively inhibit saliva and respiratory gland secretion, facilitate the clear exposure of the vocal folds during endotracheal intubation (Arany et al., 2021), and help reduce postoperative regurgitation and aspiration (Randell et al., 1991). Additionally, it can also correct the hemodynamic fluctuations induced by vagal reflexes that may occur during tracheal intubation and surgery (Stollings et al., 2014).

Glycopyrrolate has primarily been studied for its use as a preoperative agent or in combination with cholinesterase inhibitors to antagonize the muscarinic residual effects of non-depolarizing muscarinic agents (McCubbin et al., 1979; Orko and Rosenberg, 1984; Mirakhur, 1985; Mogensen et al., 1986). Studies have reported the use of glycopyrrolate in preventing the slowing down of heart rate (HR) caused by depolarizing muscarinic agents, such as succinylcholine, and preventing oculocardiac reflex during ophthalmologic surgery (Cozanitis et al., 1980; Hunsley et al., 1982; Greenan, 1984; Chisakuta and Mirakhur, 1995), mainly in adult populations (McCubbin et al., 1979; Cozanitis et al., 1980; Greenan, 1984; Orko and Rosenberg, 1984; Mirakhur, 1985; Mogensen et al., 1986). Previous studies have yielded inconsistent findings regarding whether glycopyrrolate offers advantages over other anti-cholinergic drugs, such as atropine, in terms of its effects on patients’ HR, blood pressure (BP), oral and respiratory glandular secretions, postoperative nausea and vomiting, and postoperative cognitive function (Ostheimer, 1977; Mostafa and Vucevic, 1984; Coventry et al., 1987; Simpson et al., 1987; Toft and Rømer, 1987; Grønnebech et al., 1993; Chisakuta and Mirakhur, 1995; Green et al., 2010). In the current study, we designed a clinical observational trial and aimed to compare the effects of glycopyrrolate and atropine on perioperative oral secretions, hemodynamics, and recovery quality when administered before anesthetic induction in children undergoing tonsillectomy and adenoidectomy. The study findings can provide a clinical basis for the rational selection of anti-cholinergic drugs during general anesthesia with tracheal intubation in pediatric patients.

## Materials and methods

### Study design

This clinical trial received approval from the Ethics Committee of Hangzhou First People’s Hospital, Zhejiang University School of Medicine (approval number: IIT-20220421-0062-01). This clinical trial was registered in the China Clinical Trials Registry (registration number: ChiCTR2200063578), and written informed consent was obtained from the parents of all patients.

### Participants

The inclusion criteria were as follows: age 3–10 years, weight >10 kg, absence of congenital diseases, and no symptoms of severe upper respiratory tract infection (e.g., no fever, clear discharge, and otherwise healthy) or non-infection-induced symptoms (e.g., epilepsy, myasthenia gravis, and myotonic dystrophy). The exclusion criteria included refusal to participate, history of allergy to anti-cholinergic drugs, crying before surgery, severe upper respiratory tract infections or lung infections (e.g., sneezing; nasal congestion; runny nose; cough; sputum; body temperature >38°; lethargy; or signs of lung involvement such as rales, bronchial wheezing, and diminished or absent breath sounds), acute asthma exacerbation, and abnormal preoperative blood markers (e.g., elevated white blood cells and C-reactive protein due to infection, elevated transaminases due to abnormal liver function, elevated creatinine and urea nitrogen due to abnormal renal function), abnormal prothrombin time and activated partial thromboplastin time values due to abnormal coagulation function).

A total of 103 patients with chronic tonsillitis and adenoid hypertrophy who met the inclusion criteria were included in the final analysis. The observation period of this prospective, single-center, randomized, double-blind, controlled trial was from September 2022 to December 2022.

### Determination of sample size

According to the pretest results involving a total of 50 children, the mean intraoperative HR for groups A and B were 116.77 ± 9.56 beats/min and 123.76 ± 10.96 beats/min, respectively. With bilateral α = 0.05 and β = 0.1, n = 45 was calculated using the formula n=Zα+Zβ21+1kσ2δ2,k=1. By considering a dropout rate of 0.1 (according to the formula, dropout rate = number of dropouts/total sample size), a total of 50 children were included in each group.

### Randomization

A total of 103 pediatric patients were selected, and their parents signed an informed consent form before surgery. The patients were assigned numbers from 1 to 103 on the basis of the surgical sequence. Groups A and B represented the glycopyrrolate group and atropine group, respectively. A random number list generated by QuickCalcs (GraphPad Prism8) was used for randomization. Subsequently, each number was matched with either group A or B, and these assignments were sealed in corresponding numbered envelopes kept by the study leader. The envelopes were opened upon receiving written informed consent to reveal the group assignment to the anesthesiologist, thus maintaining blinding for both the subjects and the recorders.

This clinical trial was double-blinded to ensuring that both the study subjects and the observation recorders remained unaware of the patient grouping. By using the above method, the children were randomized into two groups: group A (glycopyrrolate, n = 51) and group B (atropine, n = 52). Groups A and B received intravenous glycopyrrolate and atropine, respectively, 3 min before the induction of anesthesia.

### Pre-anesthesia preparation

The researcher visited the children 1 day before surgery to obtain informed consent. All children fasted for 8 h before surgery and refrained from drinking for 2 h prior to surgery. All children had their venous access opened and the vein detained needles retained by the ward nurse the day before surgery. Upon entering the operating room, continuous monitoring was initiated using electrocardiography, HR, pulse oxygen saturation (SpO_2_), noninvasive BP (NIBP), electroencephalographic bispectral index (BIS), and body temperature.

### Anesthesia induction

General anesthesia with tracheal intubation was performed in both groups. Group A received glycopyrrolate 0.005 mg/kg (maximum dose, 0.2 mg), whereas group B received atropine 0.01 mg/kg (maximum dose 0.5 mg). These drugs were injected 3 min before the induction of anesthesia.

The anesthetic induction protocol included midazolam 0.05 mg/kg (maximum dose 1 mg), dexamethasone 0.1 mg/kg (maximum dose 2.5 mg), propofol 3 mg/kg, sufentanil 0.5 μg/kg, and cisatracurium 0.1 mg/kg.

### Anesthesia maintenance

Anesthetic maintenance protocol included propofol 5 mg/kg/h, remifentanil 0.15 μg/kg/min, and sevoflurane 2%–3%. The ventilator parameters were tidal volume of 8–10 mL/kg, minute ventilation of 100–200 mL/kg, peak inspiratory pressure of 12–20 cm H_2_O, respiratory rate of 16–20 breaths/min (adjusted for end-expiratory CO_2_ partial pressure [35–45 mmHg]), inspiratory-to-expiratory ratio of 1:1.5, and F_i_O_2_ of 0.6. The same surgical approach was used in all children. The concentration of sevoflurane was maintained at 2%–3% to achieve a BIS value of 40–60 (Zhang et al., 2019; Zhang et al., 2022).

### Postsurgery care

After surgery, all anesthetic drugs were discontinued, and the children were transferred to the postanesthesia care unit (PACU) with the tracheal tube in place and positioned in the right lateral position. A gauze (on a non-absorbent transparent film) was placed under the corner of the mouth outside the mouth to collect oral secretions. After extubation, the mass of the gauze was weighed to calculate the amount of oral secretions (1 g ≈ 1 mL).

### Observation parameters

The HR, NIBP, SpO_2_, and BIS values were recorded at eight time points: upon entering the operating room (preoperative baseline value, T0), 3 min after glycopyrrolate or atropine was injected (T1), after completing tracheal intubation (T2), at the start of surgery (T3), at the time of tonsillectomy (T4), at the end of surgery (T5), 2 minutes after surgery (T6), and after entering the PACU (T7). Furthermore, we defined T2 to T5 as the intraoperative duration and T6 to T7 as the postoperative duration. Additionally, the duration of surgery, duration of extubation (from the end of the surgery to the removal of the tracheal tube), preoperative and postoperative temperatures, and perioperative complications (e.g., hypoxemia, laryngospasm, and agitation) were recorded.

**FIGURE 1:**
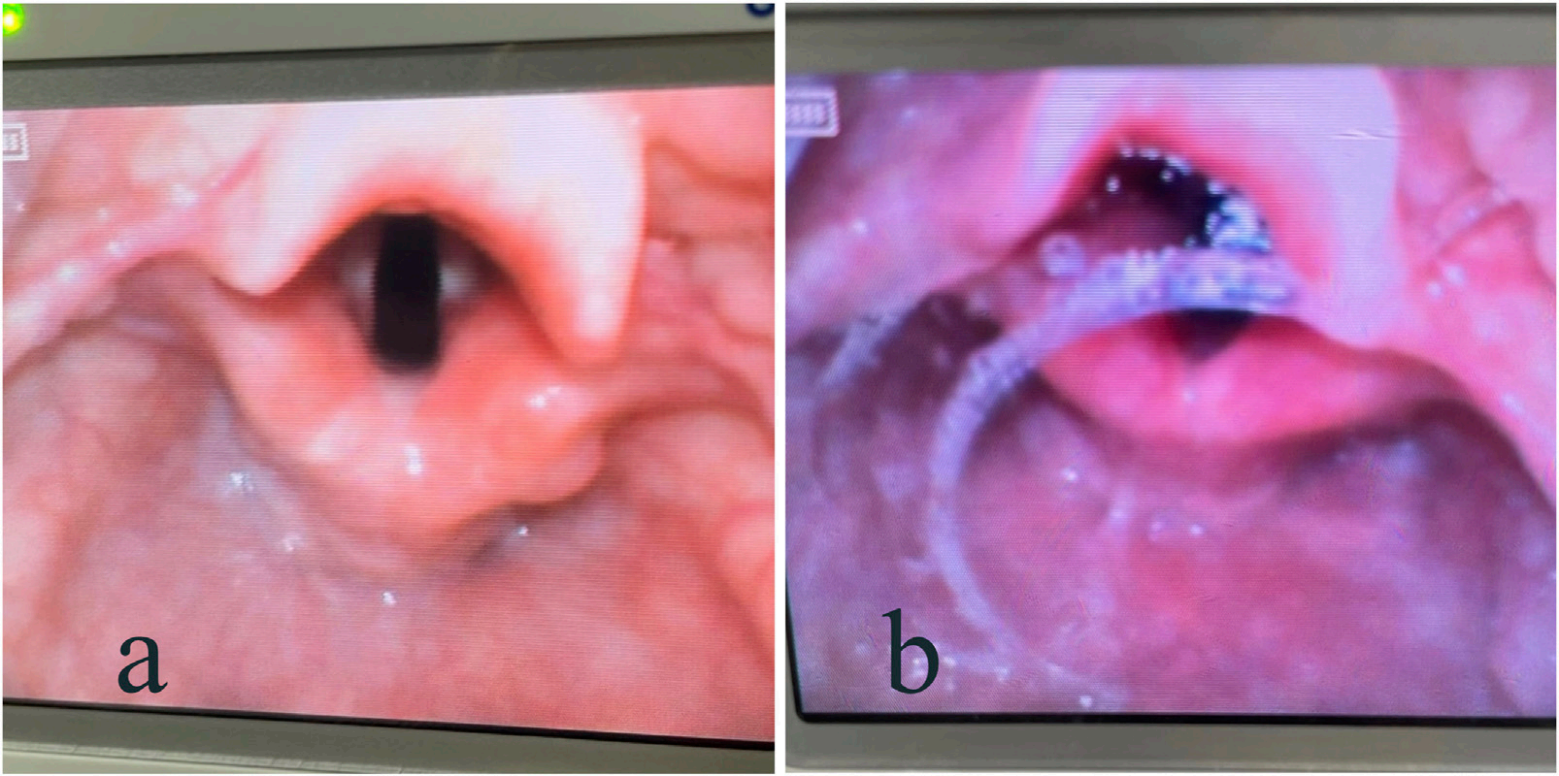
Visual laryngoscope showing secretions around the vocal cords: **(A)** dry; **(B)** wet.

The anesthesiologist assessed the secretions around the vocal cords at the time of tracheal intubation and defined them as dry or wet (Figure 1). The weight of the oral secretions (difference between the mass of the gauze placed in the corners of the mouth at the time of entry into the PACU and after extubation) was also recorded.

### Statistical analysis

Data were analyzed using SPSS version 26.0 (IBM SPSS Inc., Armonk, United States) and GraphPad Prism version 8.0. Normally distributed data (the normality of the measurements was tested using the Shapiro–Wilk method) are presented as mean ± standard deviation (x ± s). Independent samples *t*-tests were used to evaluate normally distributed continuous data, and the chi-square test was used for categorical data. A *p*-value <0.05 was considered statistically significant.

## Results

**FIGURE 2:**
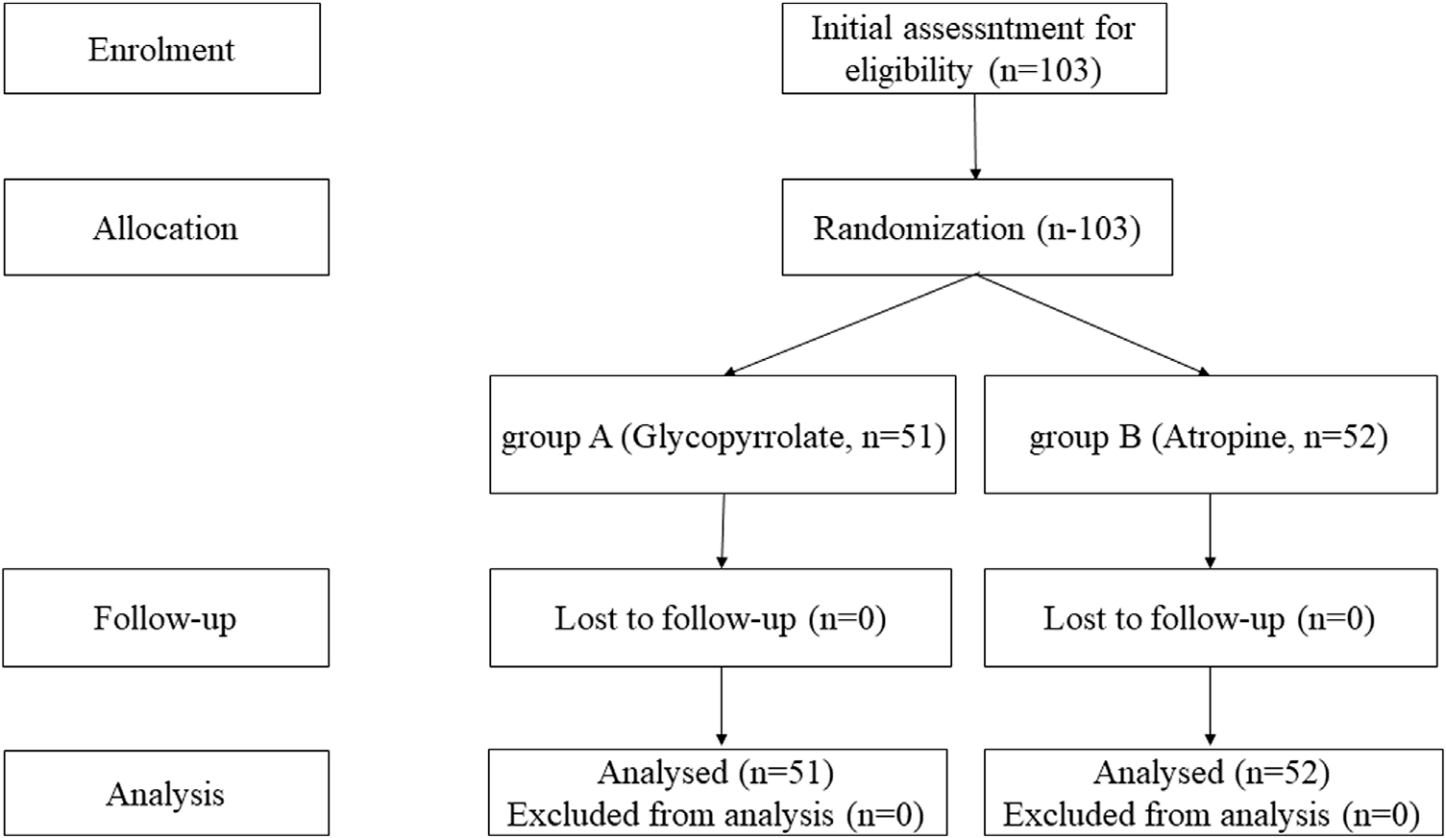
Flow diagram for the trial.

On the basis of the inclusion and exclusion criteria, a total of 103 children were recruited for this study (51 children in group A [glycopyrrolate] and 52 children in group B [atropine]). We performed statistical analysis of the data from all 103 patients and created a flow diagram for the trial (Figure 2).

### Comparison of general data between the two groups

No significant differences were observed in age, height, weight, or sex between the two groups (*p* > 0.05) (Table 1).

**TABLE 1:** Comparison of the demographic characteristics of the children between the two groups.

	Group A (GLY) (n = 51)	Group B (ATR) (n = 52)	*p*-value
Male/Female	29/22	32/20	0.629
Age-years	5.76 ± 1.93	5.44 ± 1.92	0.399
Height(cm)	117.76 ± 13.16	115.59 ± 14.50	0.427
Weight(kg)	22.24 ± 6.98	21.38 ± 7.75	0.555

Gender was tested using the chi-square or Fisher’s exact test; age, height, and weight were expressed as (mean ± standard deviation) (x ± s), and a two-sample Student’s *t*-test was used.

### Comparison of HR between the two groups

**FIGURE 3:**
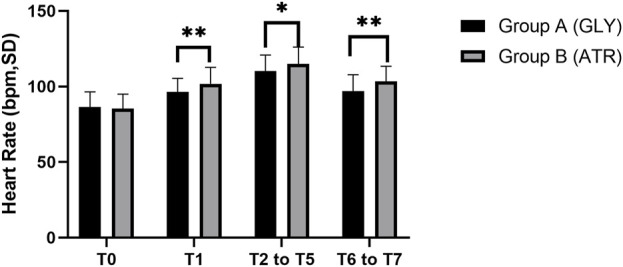
Comparison of perioperative heart rate in different time periods between the two groups T0: after entering the operation room; T1: 3 min after glycopyrrolate or atropine was injected; T2 to T5: after completing the tracheal intubation to the end of the surgery; T6 to T7: 2 min after the surgery to entering the PACU. Data were compared by using a two-sample Student’s *t*-test in groups. *p < 0.05; **p < 0.01; ***p < 0.001.

**FIGURE 4:**
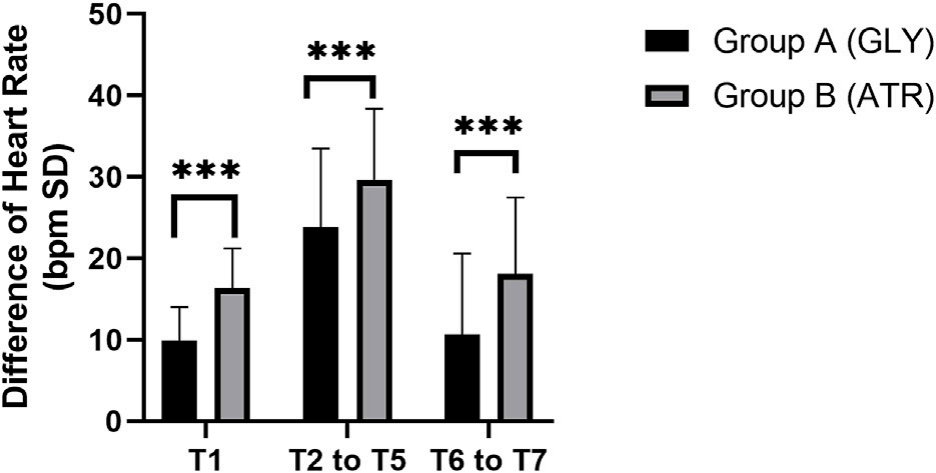
Comparison of the difference in heart rate in different time periods between the two groups. T1: 3 min after glycopyrrolate or atropine was injected; T2 to T5: after completing the tracheal intubation to the end of the surgery; T6 to T7: 2 min after the surgery to entering the PACU. Data were compared by using a two-sample Student’s *t*-test in groups. *p < 0.05; **p < 0.01; ***p < 0.001.

No significant difference was observed in the preoperative HR (T0) between the two groups. Following the administration of glycopyrrolate or atropine, the intraoperative (T2–T5) and postoperative (T6–T7) HRs of group A (glycopyrrolate) were lower than those in group B (atropine) (96.29 ± 9.11 vs. 101.85 ± 10.88, *p* = 0.006; 110.18 ± 10.58 vs. 114.94 ± 11.14, *p* = 0.028; 96.96 ± 10.81 vs. 103.38 ± 10.09, *p* = 0.002) (Figure 3). Furthermore, the difference in HR after the injection of glycopyrrolate or atropine (T1) and preoperative (T0) HR was lower in group A (glycopyrrolate) than in group B (atropine). The difference between intraoperative (T2 to T5) HR and preoperative (T0) HR was also lower in group A (glycopyrrolate) than in group B (atropine) (23.84 ± 9.62 vs. 29.65 ± 8.75, *p* = 0.002). The difference between postoperative (T6 to T7) HR and preoperative (T0) HR was lower in group A (glycopyrrolate) than in group B (atropine) (10.63 ± 9.97 vs. 18.09 ± 9.39, *p* = 0.000) (Figure 4).

**FIGURE 5:**
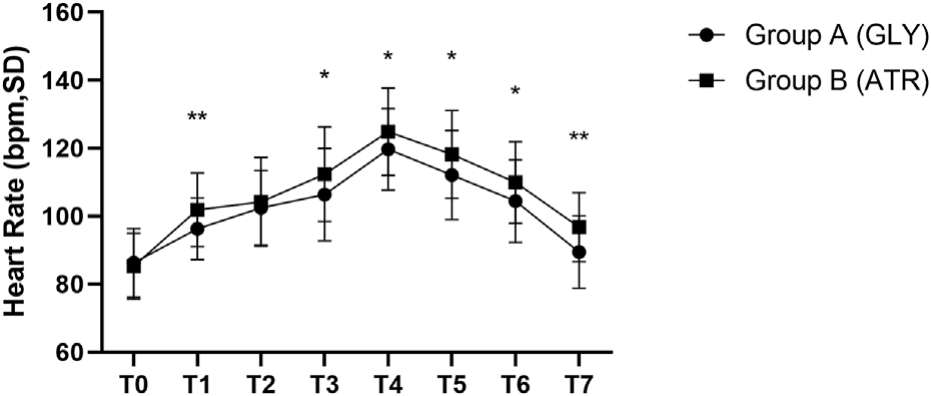
Comparison of heart rate at different time points in the perioperative period between the two groups. T0: after entering the operating room; T1: 3 min after glycopyrrolate or atropine was injected; T2: after completing the tracheal intubation; T3: at the start of the surgery; T4: at the time of tonsillectomy; T5: at the end of the surgery; T6: 2 min after the surgery; T7: after entering the PACU. Data were compared by using a two-sample Student’s *t*-test in groups. *p < 0.05; **p < 0.01; ***p < 0.001.

In addition, group A exhibited lower HRs than group B at the following time points, with statistically significant differences: T1, T3, T4, T5, T6, and T7 (96.29 ± 9.11 vs. 101.85 ± 10.88, *p* = 0.066; 106.39 ± 13.61 vs. 112.40 ± 13.92, *p* = 0.029; 119.73 ± 11.99 vs. 124.90 ± 12.90, *p* = 0.037; 112.16 ± 13.11 vs. 118.21 ± 12.88, *p* = 0.020; 104.45 ± 12.21 vs. 109.96 ± 11.99, *p* = 0.023; 89.47 ± 10.69 vs. 96.79 ± 10.15, *p* = 0.001) (Figure 5).

### Comparison of mean arterial pressure between the two groups

**FIGURE 6:**
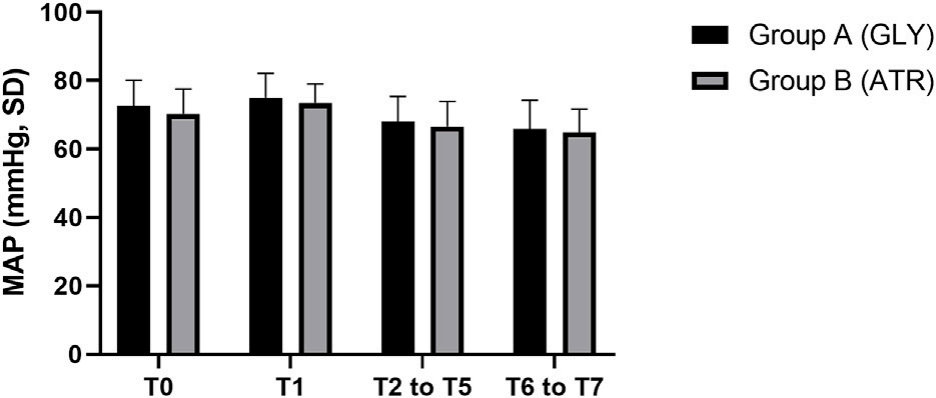
Comparison of perioperative MAP in different time periods between the two groups. MAP: mean arterial pressure; T0: after entering the operation room; T1: 3 min after glycopyrrolate or atropine was injected; T2 to T5: after completing the tracheal intubation to the end of the surgery; T6 to T7: 2 min after the surgery to entering the PACU. Data were compared by using a two-sample Student’s *t*-test in groups. *p < 0.05; **p < 0.01; ***p < 0.001.

**FIGURE 7:**
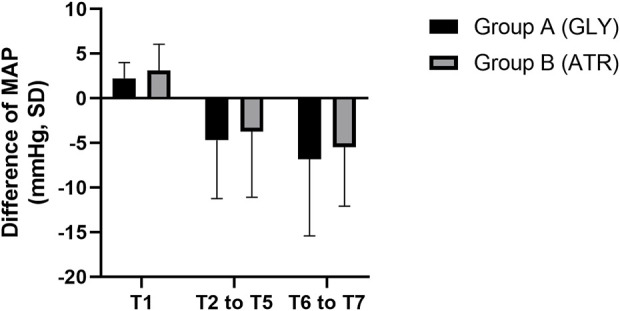
Comparison of the difference in heart rate in different time periods between the two groups. MAP: mean arterial pressure; T1: 3 min after glycopyrrolate or atropine was injected; T2 to T5: after completing the tracheal intubation to the end of the surgery; T6 to T7: 2 min after the surgery to entering the PACU. Data were compared by using a two-sample Student’s *t*-test in groups. *p < 0.05; **p < 0.01; ***p < 0.001.

**FIGURE 8:**
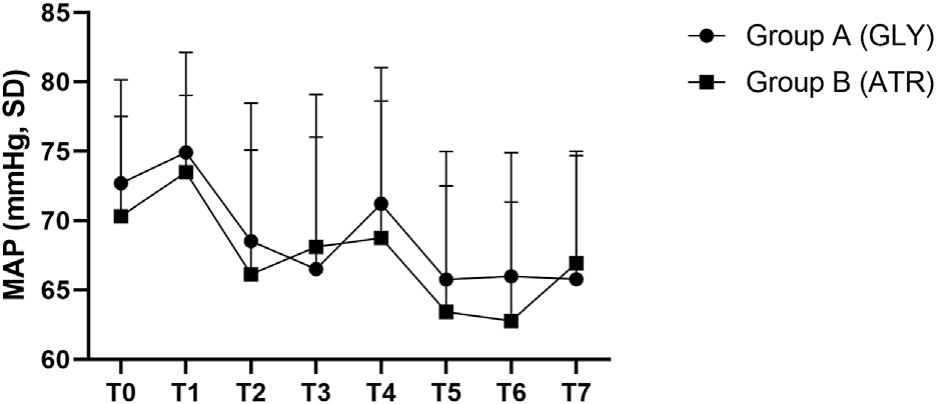
Comparison of MAP at different time points in the perioperative period between the two groups. MAP: mean arterial pressure; T0: after entering the operating room; T1: 3 min after glycopyrrolate or atropine was injected; T2: after completing the tracheal intubation; T3:at the start of the surgery; T4: at the time of tonsillectomy; T5: at the end of the surgery; T6: 2 min after the surgery; T7: after entering the PACU. Data were compared by using a two-sample Student’s *t*-test in groups. *p < 0.05; **p < 0.01; ***p < 0.001.

No significant differences were observed in mean arterial pressure (MAP) between the two groups preoperatively (T0), following the administration of glycopyrrolate or atropine (T1), intraoperatively (T2–T5), and postoperatively (T6–T7) (Figure 6). Furthermore, no significant differences were observed between the two groups in terms of the difference in MAP after the administration of glycopyrrolate or atropine (T1) and preoperative (T0) MAP. Similarly, no significant difference was observed in the intraoperative (T2 to T5) and preoperative (T0) MAPs between the two groups. There was also no significant difference between the two groups in the postoperative (T6 to T7) and preoperative (T0) MAP. (Figure 7). In addition, no significant difference was noted in MAP between the two groups of children at any time point (Figure 8).

### Comparison of SpO_2,_ duration of surgery, extubation time, and body temperature between the two groups

There were no significant differences in SpO2, duration of surgery, extubation time, or body temperature between the two groups (*p* > 0.05; Tables 2, 3).

**TABLE 2:** Comparison of pulse oxygen saturation in perioperative period between two groups.

	Group A (GLY) (n = 51)	Group B (ATR) (n = 52)	*p*-value
T0 SpO_2_	98.59 ± 1.22	98.48 ± 1.20	0.653
T1 SpO_2_	98.96 ± 0.94	98.98 ± 1.08	0.920
T2to T5 SpO_2_	99.98 ± 0.93	99.93 ± 1.05	0.789
T6 to T7 SpO_2_	99.13 ± 0.71	99.16 ± 0.66	0.790

Pulse oxygen is expressed as (mean ± standard deviation) (x ± s), and a two-sample Student’s t-test was used in groups. T0: after entering the operation room; T1: 3 min after glycopyrrolate or atropine was injected; T2 to T5: after completing the tracheal intubation to the end of the surgery; T6 to T7: 2 min after the surgery to entering the PACU.

**TABLE 3:** Comparison of the length of operation, length of extubation, and body temperature between the two groups.

	Group A (GLY) (n = 51)	Group B (ATR) (n = 52)	*p*-value
Length of operation (min)	30.10 ± 10.44	31.88 ± 9.97	0.376
Length of extubation (min)	34.10 ± 8.77	33.48 ± 8.13	0.712
Preoperative body temperature (°C)	36.48 ± 0.24	36.54 ± 0.26	0.290
Postoperative body temperature (°C)	36.51 ± 0.25	36.54 ± 0.36	0.598

Length of operation, length of extubation, preoperative body temperature, postoperative body temperature was expressed as (mean ± standard deviation) (x ± s), and a two-sample Student’s *t*-test was used in groups.

### Comparison of the weight of oral secretions and the degree of wetness around the vocal cords between the two groups

The weights of oral secretions and the degree of wetness around the vocal cords during tracheal intubation did not significantly differ between the two groups (*p* = 0.725 and 0.452, respectively; Tables 4, 5).

**TABLE 4:** Comparison of the oral secretions between the two groups.

	Group A (GLY) (n = 51)	Group B (ATR) (n = 52)	*p*-value
Oral secretions(g)	1.81 ± 1.32	1.91 ± 1.54	0.725

The oral secretions were expressed as (mean ± standard deviation) (x ± s), and a two-sample Student's *t*-test was used in groups.

**TABLE 5:** Anesthetist’s assessment of secretions around the vocal folds during tracheal intubation.

	Group A (GLY) (n = 51)	Group B (ATR) (n = 52)
Dry	44	42
Wet	7	10

*p* = 0.452.

### Comparison of perioperative complications between the two groups

No significant differences were found in perioperative complications between the two groups (Table 6). Group A had one case of postawakening agitation (2.0%), whereas group B had one case of severe cough postextubation (1.9%). No complications, such as hypoxia or laryngospasm, occurred in either group.

**TABLE 6:** Comparison of perioperative complications between the two groups.

	Group A (GLY) (n = 51)	Group B (ATR) (n = 52)	*p*-value
Postawakening agitation	1 (2.0%)	0	0.992
Postawakening cough	0	1 (1.9%)	1.000
Hypoxia	0	0	NS
Laryngospasm	0	0	NS

NS, not significant.

## Discussion

As children may have preoperative separation anxiety, severe separation anxiety will in turn lead to more crying, which will increase oral secretions and affect our experimental results. Therefore, children with good separation anxiety and no crying were included in our study.

We compared the differences in perioperative oral secretions, hemodynamics, and recovery quality with glycopyrrolate versus with atropine before anesthesia induction in children undergoing tonsillectomy and adenoidectomy.

Studies have indicated that the intravenously injected dose of atropine is approximately 2–2.5 times higher than that of glycopyrrolate (Cozanitis et al., 1980; Hunsley et al., 1982; Orko and Rosenberg, 1984; Mogensen et al., 1986; Grønnebech et al., 1993; Chisakuta and Mirakhur, 1995). Owing to this dose ratio, which we maintained in this study, we consider it to represent an equivalent dosage for both atropine and glycopyrrolate. The primary aim of the preoperative intramuscular or intravenous injection of glycopyrrolate is to reduce salivary gland, bronchial, and pharyngeal secretions (Li et al., 2021). An intramuscular injection of 0.005 mg/kg glycopyrrolate or 0.20–0.25 mg glycopyrrolate in adults before anesthesia induction aids in reducing oropharyngeal secretions, thereby improving visualization for tracheal intubation (Mirakhur et al., 1978; Cho et al., 2018). Despite several studies comparing the anti-salivary effects of glycopyrrolate and atropine, no consistent conclusions have been reached. For instance, Warran et al. (Warran et al., 1981) compared the oropharyngeal secretions (defined as oropharyngeal “moist” or “dry” depending on whether aspiration was required) during tracheal intubation, intraoperatively, and postoperatively in three groups that were given atropine (i.v), atropine (i.m) and glycopyrrolate (i.v) before anesthesia induction in children undergoing tonsillectomy and adenoidectomy. The results showed that there was no difference in oropharyngeal dryness at the time of intubation or in the postoperative period; however, glycopyrrolate was better at inhibiting gland secretion during surgery. Similarly, Lavis et al. (Lavis et al., 1980) found no disparity in postoperative dry mouth duration between glycopyrrolate and atropine in children undergoing tonsillectomy and adenoidectomy. Black et al. (Black et al., 1980) compared glycopyrrolate with atropine in combination with neostigmine in children for muscarinic antagonism, defining oropharyngeal conditions as “dry,” “moist,” and “unacceptably moist” and found that there was no statistical difference in the anti-salivary effect of the two drugs even though the attending anesthetist mainly believed that glycopyrrolate was more effective. In the current study, we quantitatively measured the weights of secretions collected on a gauze to evaluate the comparative anti-salivation effects of the two drugs. Our innovative approach provided quantitative data rather than subjective evaluations. All other parts of the sentence are redundant. We found that there was no significant difference in postoperative oral secretions between the two groups, and this finding is consistent with previous studies. However, our results may also be influenced by the timing of the secretion collection. We measured oral secretions collected from the moment the child entered the PACU. Prior to this, the surgeon performed adequate hemostasis and aspiration of the oropharynx. Therefore, the oropharynx of children entering the PACU was relatively clean. After surgery, without stimulation by the surgical operation, the glandular secretion may not have been as active as before and during surgery, and the children did not complain of significant dry mouth after the operation. Therefore, the results of this trial showed no significant difference in the perioperative anti-salivary effects of glycopyrrolate and atropine during pediatric tonsillectomies and adenoidectomies.

Although our primary focus was on the differing hemodynamic effects of glycopyrrolate and atropine, we found no significant differences in their perioperative anti-salivary effects during pediatric tonsillectomies and adenoidectomies. Notably, the HR of group A was significantly lower than that of group B after drug administration both intraoperatively and postoperatively. The HR of group A fluctuated less after drug administration, both intraoperatively and postoperatively, and remained close to the baseline level for a longer period. This result was similar to that reported by Desalu et al. (Desalu et al., 2005). They compared the hemodynamic effects of atropine and glycopyrrolate during general anesthesia with tracheal intubation in children and found that the mean HR increased by 35.7% from baseline in the atropine group and 22.5% in the glycopyrrolate group, with statistically significant increases of 9.7% and 13.2% in the two groups, respectively. Children have a higher HR than adults because of their high metabolism and sympathetic excitability; however, parasympathetic excitation, anesthetic overdose, or tissue hypoxia can lead to bradycardia and a severe reduction in cardiac output (Deng 2024). Preoperative anti-cholinergic agents in pediatric patients are effective in preventing perioperative bradycardia and reducing the incidence of other arrhythmias. However, prolonged periods of excessively high HRs can increase myocardial oxygen consumption. Glycopyrrolate is a selective M-receptor antagonist that is three to five times more selective for M3 and M1 receptors than for M2 (Haddad et al., 1999), which is located mainly in the heart and regulates HR. Therefore, glycopyrrolate is better able to maintain a steady HR than atropine, which has a modest effect on the increase in HR in children. In addition, there was no significant difference in the intraoperative and postoperative BPs between the two groups, similar to the results of Annila et al. (Annila et al., 1998). They compared the effects of atropine, glycopyrrolate, and saline on cardiovascular function in children undergoing general anesthesia for adenoidectomy and found that the differences in the incidence of arterial systolic pressure among the three groups were not statistically significant.

Further analysis revealed that the overall HR values in group A were lower than those in group B. Despite the HRs in both groups gradually increasing over time, those in group A were always lower than those in group B. The differences in HR observed at T1 may be related to differences in the pharmacokinetics and pharmacodynamics of atropine and glycopyrrolate. The peak of action of glycopyrrolate was slightly later than that of atropine, which may explain why the HR of children in the glycopyrrolate group was lower than that in the atropine group at T1. In addition, although the HRs of the children in both groups decreased when the surgery was completed, the HR of the children in Group A was close to the preoperative baseline HR at the time of admission to the PACU, whereas the HR of the children in the atropine group was still higher than the baseline value. This result is similar to those of Parlow (Parlow et al., 1997) et al. in healthy volunteers. They compared the parasympathetic depressant effects of atropine and glycopyrrolate and found that the time for the HR to return to baseline values was approximately double in the atropine group compared to the glycopyrrolate group. Atropine results in longer parasympathetic depression than an equivalent dose of glycopyrrolate, thus making its use in early postoperative high-risk patients (e.g., elderly patients with poor cardiopulmonary function) undesirable.

Green et al. (Green et al., 2010) found that the use of glycopyrrolate during ketamine sedation was more likely to result in postoperative nausea and vomiting in children than atropine. This concern was not observed in our study possibly because of the prophylactic dose of dexamethasone administered during induction. Dexamethasone and propofol are known to have anti-emetic effects (Tramèr et al., 1997; De Oliveira et al., 2013; Matsuura et al., 2016).

Theoretically, the CNS-related adverse effects of glycopyrrolate should be lower than those of atropine and scopolamine because the quaternary ammonium group of glycopyrrolate limits its passage across the blood–brain barrier. Previous studies have mostly focused on adult or elderly patients even though the conclusions have varied. Simpson et al. (Simpson et al., 1987) compared the effects of glycopyrrolate and atropine on orientation, attention, and short-term visual memory after general anesthesia in adults and found that postoperative short-term memory impairment occurred in the atropine group; by contrast, no significant cognitive impairment was found in the glycopyrrolate group. Sheref et al. (S Sheref, 1985) compared the recovery from anesthesia in patients after reversal of the muscle-relaxing effect of glycopyrrolate and atropine combined with neostigmine and found faster awakening in the glycopyrrolate group. Malling et al. (Malling et al., 1988) compared the combination of glycopyrrolate and atropine with neostigmine to antagonize residual muscle relaxation during hip arthroplasty in elderly patients undergoing general anesthesia and found no difference in postoperative awakening time between the two groups. In this trial, although one case of postarousal agitation occurred in group A (glycopyrrolate), no significant difference was observed in the overall time to arousal or quality of arousal between the two groups, thus further confirming the findings of Malling (Malling et al., 1988) et al. In addition, the doses of both glycopyrrolate and atropine were relatively conserved in our experiment, and no significant effects on the patients’ body temperature were found, similar to the findings of Warran et al. (Warran et al., 1981).

This study has some limitations. First, although the surgical approaches were the same, the surgeons differed, potentially introducing slight differences in their surgical practices. Second, the subjective assessment of secretions around the vocal cords at the time of intubation to compare the anti-sialagogue properties of glycopyrrolate and atropine introduces a risk of bias. The variability in grading by different anesthesiologists may distort the results systematically, inviting criticism. To eliminate this interoperative variability, future trials should designate a single expert anesthesiologist to grade all vocal cord photographs. Finally, there was a lack of rigorous and comprehensive postoperative assessment covering cognitive function, learning ability, and memory. Although it is challenging for children to self-assess these areas independently, future studies should address these limitations for more robust conclusions.

## Conclusion

Glycopyrrolate offers smoother intraoperative and postoperative HR changes and less volatility than atropine, with no adverse effect on BP or postoperative arousal. It effectively inhibits oral secretions and stabilizes hemodynamics, thus making it a suitable alternative to atropine for anesthesia induction in pediatric tonsil and adenoid surgeries.

## Data Availability

The raw data supporting the conclusion of this article will be made available by the authors, without undue reservation.
